# Effects of hydroxyethyl starch and gelatin on the risk of acute kidney injury following orthotopic liver transplantation: A multicenter retrospective comparative clinical study

**DOI:** 10.1515/med-2020-0183

**Published:** 2021-02-23

**Authors:** Yingqi Chen, Xinyu Ning, Haiyang Lu, Sainan Zhu, Anshi Wu, Jia Jiang, Shanshan Mu, Jing Wang, Xu Niu, Shengnan Li, Lingdi Hou, Yanxing Zhao, Wenfei Lv, Meixia Shang, Chen Yao, Shujun Han, Ping Chi, Fushan Xue, Yun Yue

**Affiliations:** Department of Anesthesiology, Beijing Chaoyang Hospital, Capital Medical University, No. 8 Gongtinan Road, Chaoyang District, Beijing, 100020, China; Department of Anesthesiology, The Third Medical Centre, Chinese People’s Liberation Army General Hospital, No. 69 Yongding Road, Haidian District, Beijing, 100000, China; Department of Anesthesiology, Beijing Friendship Hospital, Capital Medical University, No. 95 Yongan Road, Xicheng District, Beijing, 100000, China; Department of Biostatistics, Peking University First Hospital, Beijing, 100034, China; Department of Anesthesiology, Beijing You’An Hospital, Capital Medical University, No. 8 Xitou Road, You’an Menwai, Fengtai District, Beijing, 100069, China

**Keywords:** hydroxyethyl starch, gelatin, acute kidney injury, liver transplantation, risk factors

## Abstract

**Objectives:**

This multicenter retrospective study aimed to compare the effects of HES and gelatin (GEL) on the risk of post-OLT AKI.

**Method:**

A total of 1,672 patients undergoing OLT were enrolled from major transplant centers in China between 2005 and 2013. These patients were divided into three groups: GEL, hydroxyethyl starch (HES), and GEL + HES group.

**Results:**

There was no significant difference in the incidence of post-OLT AKI among the GEL, HES, and GEL + HES groups. The GEL + HES group had a lower incidence of stage II post-OLT AKI than the other two groups. Compared with patients receiving GEL, patients receiving HES did not harbor an increased risk of AKI. Our results showed that MELD score (adjusted odds ratio [OR], 1.579; 95% confidence interval [CI], 1.123–2.219; *P* = 0.009) and preoperative anemia (adjusted OR, 1.533; 95% CI, 1.212–1.939; *P <* 0.001) were independent risk factors for post-OLT AKI, and normal preoperative Scr level (vs abnormal; adjusted OR, 0.402; 95% CI, 0.222–0.729; *P* = 0.003) was independent protective factors for post-OLT AKI.

**Conclusion:**

This large-scale multicenter retrospective study found that the intraoperative use of HES did not increase the overall incidence of post-OLT AKI in patients when compared with GEL, and whether to increase the risk of post-OLT AKI needs to be further explored.

## 1 Introduction

Hydroxyethyl starch (HES) is a plasma substitute widely used for intravascular volume supplement or resuscitation during transplant surgeries [1,2,3,4]. Several large-scale randomized controlled trials indicated that HES might impair kidney functions and increase the risk of acute kidney injury (AKI) in critically ill patients [5,6,7]. Intravenous human albumin and synthetic colloids are usually mandatory in patients undergoing orthotopic liver transplant (OLT) due to preoperative hypoalbuminemia and low colloid osmotic pressure [8,9]. In China, a combination of albumin and synthetic colloids (HES and/or gelatin [GEL]) has been widely used during OLT [10,11]. In addition, AKI is a common postoperative complication of OLT, and its incidence was reported to be more than 50% in post-OLT patients; AKI is associated with prolonged hospitalization and poor prognosis [12,13].

The etiology of post-OLT AKI is multifactorial [14], and the definitive mechanism regarding the potential nephrotoxicity of HES has not been well elucidated [15]. Foremost, the effects of HES on the risk of AKI remain controversial. A report from a large database including 44,176 adult patients undergoing noncardiac surgery showed that HES had dose-dependent nephrotoxicity with an odds ratio of 1.21 (95% CI, 1.06–1.38) for the development of postoperative AKI [16]. Some scholars proposed that, with the continuous upgrading of HES, its potential damage to renal function and coagulation function has been significantly reduced [17,18]. The CRISTAL randomized trial demonstrated that the use of colloids vs crystalloids does not result in significant differences in 28-day mortality and the need for renal replacement therapy in critically ill patients with hypovolemia [19]. A recent systematic review and meta-analysis of randomized controlled trials also indicated that HES, compared with crystalloid or other colloidal solutions, may not increase the risk of renal dysfunction in postoperative patients [20]. In post-OLT patients, a small-sample prospective, randomized, controlled clinical trial showed that the intraoperative use of HES as an alternative to human albumin resulted in the equivalent renal outcomes [8]. However, another retrospective clinical study found that the intraoperative use of HES significantly increased the risk of post-OLT AKI when compared with albumin, with an odds ratio of 2.94 (95% CI, 1.13–7.7) [11]. To the best of our knowledge, there has been no large-sample, multicenter clinical study evaluating the nephrotoxicity of HES. This study was aimed to compare the effects of HES and GEL on the risk of AKI after OLT.

## 2 Materials and methods

### 2.1 Patients

This multicenter retrospective study has been registered at the Chinese Clinical Trial Register (http://www.chictr.org.cn; identifier: ChiCTR-TRC-14004211). This study was approved by the Institutional Review Board of Beijing Chaoyang Hospital (No. 2013-159). A total of 1,672 patients undergoing OLT were enrolled from three major transplant centers in China (Beijing Chaoyang Hospital, Chinese People’s Liberation Army General Hospital, and Beijing You’An Hospital) between 2005 and 2013. The medical records of these patients were collected and analyzed.

Inclusion criterion was as follows: patients receiving OLT.

Exclusion criteria were as follows: (1) <14 years of age, (2) simultaneous dual-organ (liver and kidney) transplant, (3) no administration of intraoperative GEL or HES, or (4) and incomplete perioperative data.

**Figure 1:**
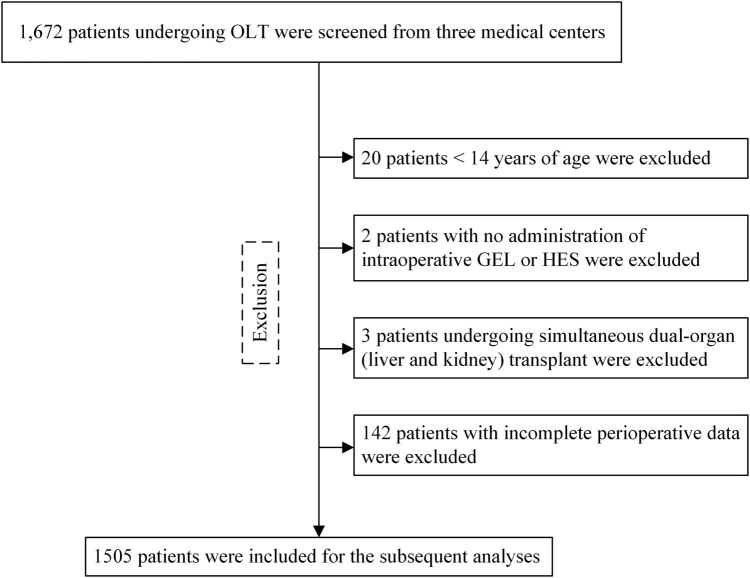
Enrollment flowchart.


Eventually, 167 patients were excluded, and 1,505 patients were included for the subsequent analyses (Figure 1). According to the types of synthetic colloids used during the OLT surgery, these patients were divided into three groups: GEL group, HES group, and GEL + HES group. Furthermore, we assessed the effects of HES exposure on the risk of post-OLT AKI; based on the volume of HES used, patients were classified into three groups: no HES exposure, low (≤1,500 mL) HES exposure, and high (>1,500 mL) HES exposure.

**Ethics approval and consent to participate:** This study has been registered at Chinese Clinical Trial Register (http://www.chictr.org.cn; Identifier: ChiCTR-TRC-14004211. Registered on 26 January 2014). The study has obtained approval from the Ethical Committee (No. 2013-159). Informed consent of patients was waived because this study was retrospective.

### 2.2 Evaluation of post-OLT AKI

The individual kidney function was evaluated within 7 days after OLT. AKI, determined based on the postoperative serum creatinine (Scr) level, was diagnosed according to the Kidney Disease Improving Global Outcomes (KDIGO) criteria [21,22]: (1) stage I, an increase in the Scr level of ≥26 μmol/L within 48 h or an Scr level of 1.50–1.99 times the baseline level within 7 days; (2) stage II, an Scr level of 2.00–2.99 times the baseline level within 7 days; and (3) stage III, an Scr level of ≥354 μmol/L or an Scr level of >3.0 times the baseline level within 7 days or the initiation of renal replacement therapy (RRT; including hemodialysis and hemofiltration). The baseline Scr level was measured on admission. In addition, the 28-day mortality and severe cases treated with RRT were analyzed.

### 2.3 Statistical analysis

The EpiData software (version 3.0, www.epidata.dk) was used for data management, and the SAS software (version 9.4, SAS Institute, Cary, North Carolina) was used for statistical analyses. Measurement variables with normal distribution were presented as means ± standard deviation (SD); nonnormal variables were presented as median (interquartile range [IQR]). Student *t*-test or Wilcoxon rank-sum test was used for two-group comparisons. One-way analysis of variance or Kruskal–Wallis test was used for three-group comparisons. Categorical variables were presented as frequency and percentage, and they were compared using the Chi-square test. The ranked variables were presented as frequency and percentage and compared using the Wilcoxon rank-sum test or Kruskal–Wallis test. The paired *t*-test or Wilcoxon paired signed-rank test was used for intragroup comparisons.

Multivariate logistic regression models were used for identifying the potential risk factors of AKI. In model 1, the candidate factors included age, gender, hemoglobin, anemia, hypoalbuminemia, inotropic agents, surgical types, the Scr level, model for end-stage liver disease (MELD) score, duration of anhepatic phase, and the use of colloids (GEL, HES, or GEL + HES). In model 2, besides the aforementioned factors, the HES exposure was also included for the analysis. The odds ratios (ORs) and 95% confidence intervals (CIs) were calculated in the two models. Two-tailed *P <* 0.05 was considered statistically significant.

## 3 Results

### 3.1 Clinical characteristics

There were 736 patients in the GEL group, 586 patients in the HES group, and 183 patients in the GEL + HES group. The detailed clinical characteristics were summarized in Table 1. There were no significant differences in gender or age among the three groups. The GEL + HES group has the highest hemoglobin level (112.0 ± 27.3 g/L) and preoperative Scr level [69.5 (57.9, 84.5) μmol/L] and the lowest MELD score (10.9 ± 8.4) than the GEL and HES groups (hemoglobin level, *P* < 0.001; preoperative Scr level, *P* = 0.041; MELD score, *P* < 0.001). In the GEL group, 603 (82.0%) patients underwent classic OLT and 133 (18.0%) patients underwent piggyback OLT; in the HES group, 350 (59.7%) patients underwent classic OLT and 236 (40.3%) patients underwent piggyback OLT; and in the GEL + HES group, 96 (52.5%) patients underwent classic OLT and 87 (47.5%) patients underwent piggyback OLT. The GEL group had the highest proportion of patients with vasopressin use (80.2%) and patients with diuretic use (77.2%) than other groups (vasopressin use, *P* = 0.007; diuretic use, *P* < 0.001). There was no significant difference in immunosuppressant use (*P* = 0.825) or surgery duration (*P* = 0.447) among the three groups. The anhepatic phase was longest in the GEL + HES group (*P* < 0.001).

**Table 1:** Clinical characteristics of enrolled subjects

Characteristics	GEL group (*n* = 736)	HES group (*n* = 586)	GEL + HES group (*n* = 183)	*P* value
Male, *n* (%)	606 (82.3)	470 (80.2)	158 (86.3)	0.160
Age (years)	49.4 ± 9.6	49.2 ± 10.2	49.0 ± 8.9	0.924
Hemoglobin (g/L)	104.2 ± 24.0	103.2 ± 24.2	112.0 ± 27.3	<0.001
Preoperative Scr (μmol/L)	65.0 [53.0, 82.0]	65.0 [53.0, 79.6]	69.5 [57.9, 84.5]	0.041
MELD score	13.8 ± 9.3	12.0 ± 9.6	10.9 ± 8.4	<0.001
**Type of OLT,** ***n*** **(%)**				
Classic	603 (82.0)	350 (59.7)	96 (52.5)	<0.001
Piggyback	133 (18.0)	236 (40.3)	87 (47.5)	
Vasopressin use, *n* (%)	590 (80.2)	439 (74.9)	129 (70.5)	0.007
Diuretic use, *n* (%)	568 (77.2)	352 (60.1)	81 (44.3)	<0.001
Immunosuppressant use, *n* (%)	674 (91.6)	533 (91.0)	169 (92.3)	0.825
Surgery duration (hours)	10.1 ± 2.4	10.1 ± 2.7	10.2 ± 2.3	0.477
Anhepatic phase (minutes)	67.8 ± 27.3	75.6 ± 28.9	84.0 ± 34.7	<0.001

Abbreviations: GEL, gelatin; HES, hydroxyethyl starch; Scr, serum creatinine; OLT, orthotopic liver transplantation; MELD, model for end-stage liver disease.

### 3.2 Impacts of HES on postoperative renal function and outcomes

**Figure 2:**
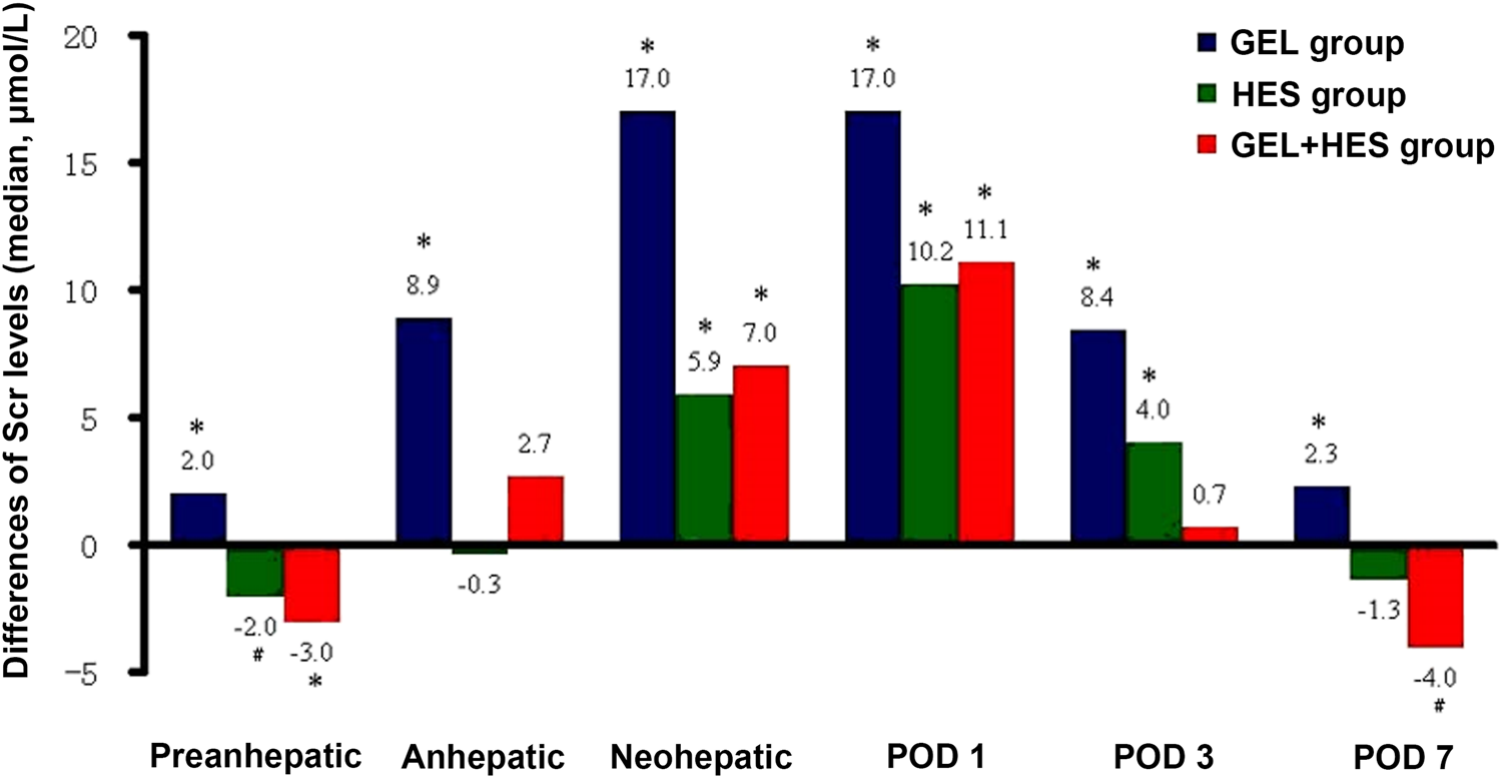
Comparisons of intraoperative and postoperative Scr levels (μmol/L) with the preoperative baseline levels. POD: postoperative day; ^*^ *P* < 0.001, ^#^ *P* < 0.05, compared with the preoperative baseline levels.

The comparisons between postoperative Scr levels and preoperative baseline levels are shown in Figure 2. On day 1 postoperatively, the Scr levels were significantly increased in all groups when compared with the baseline levels (*P* < 0.001), and the increase of Scr levels was most remarkable in the GEL group (*P* < 0.001). On day 3 and day 7 postoperatively, the Scr levels were significantly decreased than those on day 1 postoperatively (*P* < 0.001). On postoperative day 7, the Scr levels were lower than the baseline levels in the HES and GEL + HES groups (*P* < 0.001), and they were higher than the baseline levels in the GEL group (*P* < 0.001).

**Figure 3:**
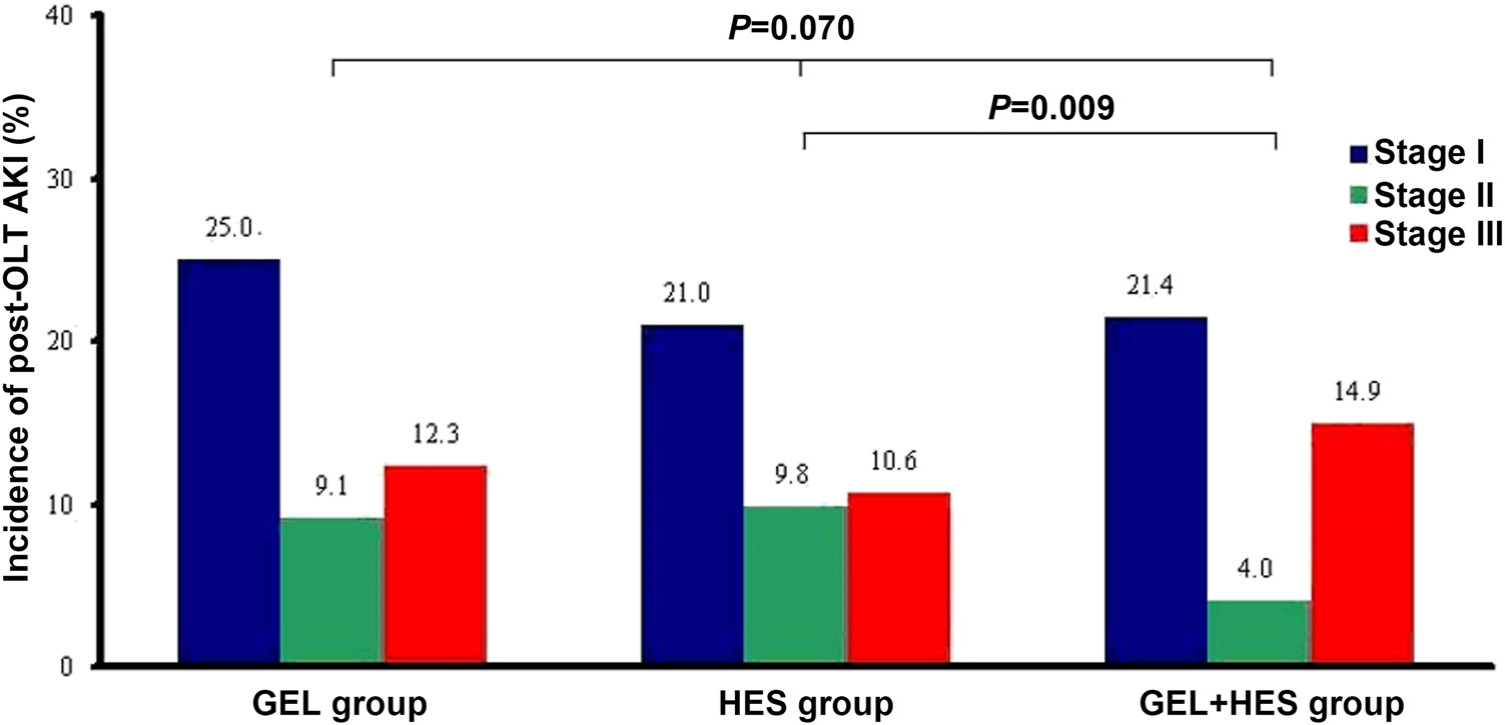
Incidence and stages of post-OLT AKI in different groups.

The overall incidence of post-OLT AKI was 46.5% in the GEL group, 41.5% in the HES group, and 40.4% in the GEL + HES group (Figure 3). There was no significant difference in the overall incidence of post-OLT AKI among the three groups (*P* = 0.119). The incidences of stages I, II, and III AKI were 26.9, 9.8, and 13.3% in the GEL group, respectively; 23.0, 10.8, and 11.6% in the HES group, respectively; and 23.5, 4.4, and 16.4% in the GEL + HES group, respectively. The GEL + HES group had a lower incidence of stage II post-OLT AKI than the other two groups (*P* = 0.035). There were no significant differences in the postoperative 28-day mortality (*P* = 0.538) or administration of RRT among the three groups (*P* = 0.417).

**Figure 4:**
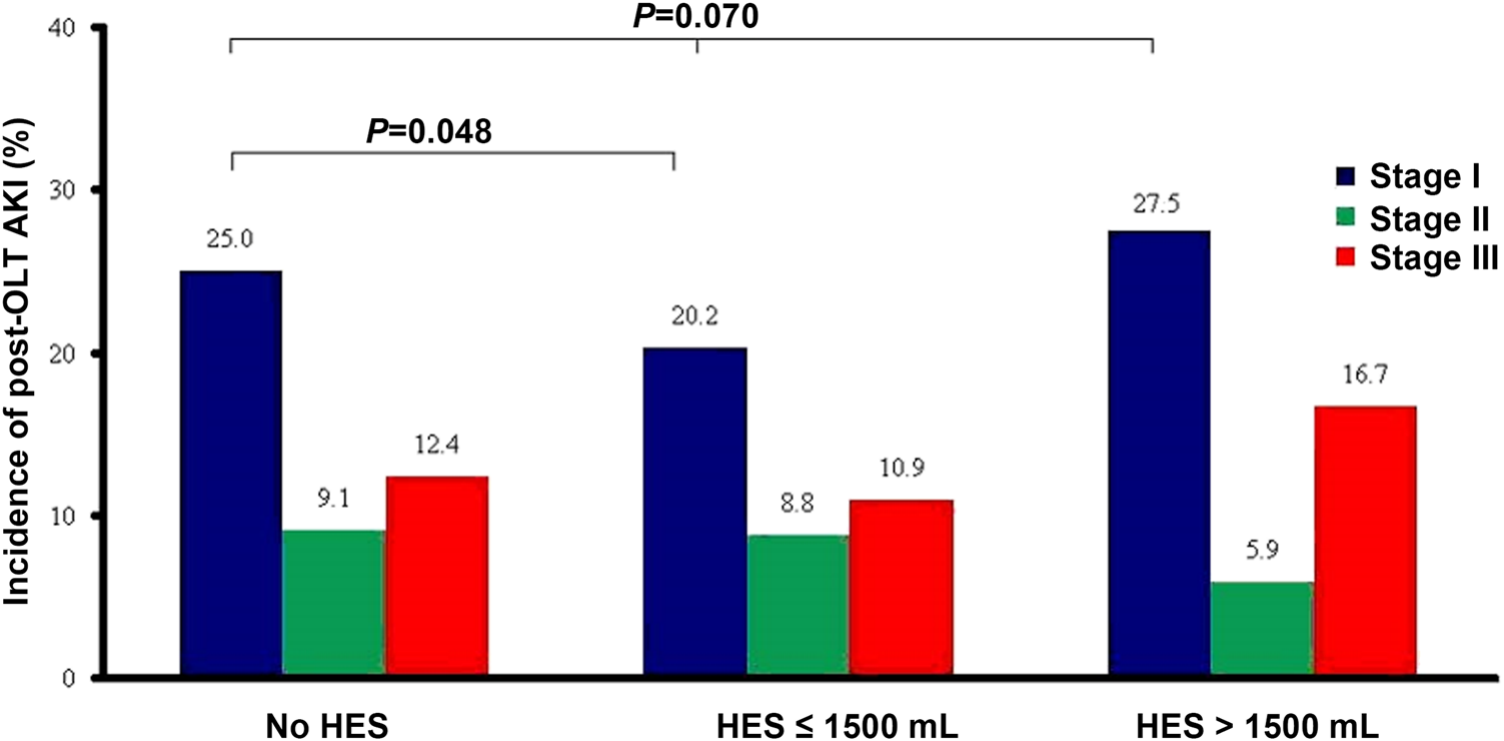
Incidence and stages of post-OLT AKI in patients with different HES exposures.

The overall incidence of post-OLT AKI in patients with no HES exposure, low HES exposure, and high HES exposure was 47.6, 42.3, and 40.4%, respectively (Figure 4). There was no significant difference in the HES exposure between patients with AKI and patients without AKI (*P* = 0.070). The postoperative data are summarized in Table 2.

**Table 2:** Postoperative outcomes

Outcome parameters	GEL group (*n* = 736)	HES group (*n* = 586)	GEL + HES group (*n* = 183)	*P* value
**Scr (μmol/L)**				
Postoperative day 1	82.0 [62.0, 112.3]	76.5 [60.0, 105.0]	84.9 [66.1, 114.0]	0.007
Postoperative day 3	73.0 [57.0, 103.0]	68.8 [55.0, 94.0]	77.0 [57.0, 97.7]	0.027
Postoperative day 7	67.0 [55.0, 88.0]	63.0 [52.0, 84.0]	66.0 [57.2, 85.0]	0.008
Recovery time (hours)	7.3 [5.0, 12.0]	7.0 [4.5, 11.0]	8.0 [5.3, 22.5]	0.008
Ventilation time (hours)	10.5 [7.5, 16.2]	14.0 [8.7, 30]	12.8 [9.3, 29.5]	<0.001
**Fluid input (mL)**				
Postoperative day 1	3494.7 ± 1539.72	3628.1 ± 1673.3	3481.6 ± 1626.7	0.318
Postoperative day 3	3325.9 ± 1428.0	3665.1 ± 1619.2	3134.1 ± 1222.8	<0.001
Postoperative day 7	2786.2 ± 1625.1	3096.1 ± 1737.2	2486.6 ± 1238.4	<0.001
**Urine output (mL)**				
Postoperative day 1	2577.2 ± 1222.8	2367.9 ± 1132.3	2324.9 ± 1062.9	<0.001
Postoperative day 3	2632.9 ± 1190.0	2847.5 ± 1214.8	2615.8 ± 951.1	<0.001
Postoperative day 7	2442.1 ± 1080.8	2643.5 ± 1216.8	2533.0 ± 1040.6	0.006
**AKI,** ***n*** **(%)**				
Total	342 (46.5)	243 (41.5)	74 (40.4)	0.119
Stage I	198 (26.9)	135 (23.0)	43 (23.5)	0.241
Stage II	72 (9.8)	63 (10.8)	8 (4.4)	0.035
Stage III	98 (13.3)	68 (11.6)	30 (16.4)	0.231
28-day mortality, *n* (%)	26 (3.5)	16 (2.7)	4 (2.2)	0.538
RRT, *n* (%)	39 (5.3)	38 (6.5)	14 (7.7)	0.417
Hemodialysis	7 (1.0%)	8 (1.4%)	2 (1.1%)	0.777
Hemofiltration	32 (4.3%)	30 (5.1%)	12 (6.6%)	0.446
MELD score	13.76 ± 9.28	11.97 ± 9.60	10.87 ± 8.38	<0.001
**MELD group**				
Low score group (<30), *n* (%)	684 (86.80)	571 (90.78)	181 (91.88)	0.023
High score group (≥30), *n* (%)	104 (13.20)	58 (9.22)	16 (8.12)	
**Time of anhepatic phase**				
≤60 min, *n* (%)	458 (57.76)	269 (42.30)	64 (31.84)	<0.001
>60 min, *n* (%)	335 (42.24)	367 (57.70)	137 (68.16)	

Abbreviations: GEL, gelatin; HES, hydroxyethyl starch; Scr, serum creatinine; AKI, acute kidney injury; RRT, renal replacement treatment.

Meanwhile, we also evaluated MELD score to study the renal function more comprehensively. The numbers of post-OLT AKI patients were 788 in the GEL group, 629 in the HES group, and 197 in the GEL + HES group (*P* < 0.001). The mean values of the MELD score were 13.76 ± 9.28 in the GEL group, 11.97 ± 9.60 in the HES group, and 10.87 ± 8.38 in the GEL + HES group. The MELD scores were divided into high and low groups by 30 points. The incidence of the low-score groups was 86.80% in the GEL group, 90.78% in the HES group, and 91.88% in the GEL + HES group (*P* = 0.023). As for the duration of the anhepatic phase, the incidence of the part ≤60 min was 57.76% in the GEL group, 42.30% in the HES group, and 31.84% in the GEL + HES group (*P* < 0.001).

### 3.3 Risk factors for post-OLT AKI

A total of 671 (44.6%) patients developed post-OLT AKI, and univariate regression analysis showed that the preoperative Scr level, hemoglobin level, MELD score, and hypoalbuminemia might be potential risk factors of post-OLT AKI (*P* < 0.05). However, age, gender, types of OLT, use of vasopressin, duration of anhepatic phase, type of colloidal solution, and dosage of intraoperative HES were not significantly associated with the occurrence of post-OLT AKI (*P* > 0.05). The detailed data are summarized in Table 3.

**Table 3:** Univariate analysis for risk factors of post-OLT AKI

Variables	Non-AKI (*n* = 834)	AKI (*n* = 671)	*P* value
Age (years)	49.3 ± 9.6	49.2 ± 9.9	0.829
**Gender**			
Male, *n* (%)	691 (82.9)	543 (80.9)	0.333
Female, *n* (%)	143 (17.1)	128 (19.1)	
**Preoperative Scr level (μmol/L)**			
Normal, *n* (%)	817 (98.0)	638 (95.1)	0.002
Abnormal, *n* (%)	17 (2.0)	33 (4.9)	
Hemoglobin (g/L)	107.1 ± 24.9	101.9 ± 24.2	<0.001
**MELD score**			
<25, *n* (%)	763 (91.5)	577 (86.0)	0.001
≥25, *n* (%)	71 (8.5)	94 (14.0)	
**Hypoalbuminemia**			
Present, *n* (%)	560 (67.2)	415 (61.9)	0.032
Absent, *n* (%)	274 (32.8)	256 (38.1)	
**Types of OLT**			
Classic, *n* (%)	589 (70.6)	460 (68.6)	0.385
Piggyback, *n* (%)	245 (29.4)	211 (31.4)	
**Vasopressin**			
Use, *n* (%)	635 (76.1)	523 (77.9)	0.409
None, *n* (%)	199 (23.9)	148 (22.1)	
**Anhepatic phase**			
≤60 min, *n* (%)	419 (50.2)	332 (49.5)	0.769
>60 min, *n* (%)	415 (49.8)	339 (50.5)	
**Type of colloidal solution**			
GEL, *n* (%)	386 (46.3)	350 (52.2)	
HES, *n* (%)	339 (40.7)	247 (36.8)	0.070
GEL + HES, *n* (%)	109 (13.1)	74 (11.0)	
**HES dosage,** ***n*** **(%)**			
None	386 (46.3)	350 (52.2)	
≤1,500 mL	339 (40.7)	247 (36.8)	0.070
>1,500 mL	109 (13.0)	74 (11.0)	

Abbreviations: AKI, acute kidney injury; Scr, serum creatinine; MELD, model for end-stage liver disease; OLT, orthotopic liver transplantation; GEL, gelatin; HES, hydroxyethyl starch.

Further multivariate logistic regression analysis showed that MELD score (adjusted odds ratio [OR], 1.579; 95% confidence interval [CI], 1.123–2.219; *P* = 0.009) and preoperative anemia (adjusted OR, 1.533; 95% CI, 1.212–1.939; *P <* 0.001) were independent risk factors for post-OLT AKI, and normal preoperative Scr level (vs abnormal; adjusted OR, 0.402; 95% CI, 0.222–0.729; *P* = 0.003) was independent protective factors for post-OLT AKI. The detailed statistical results are summarized in Table 4.

**Table 4:** Multivariate logistic regression analysis for risk factors of post-OLT AKI

Covariates	Adjusted OR	95% CI	*P* value
MELD score (high vs low)	1.579	1.123–2.219	0.009
Anemia (with vs without)	1.533	1.212–1.939	<0.001
Hypoalbuminemia (with vs without)	1.130	0.903–1.414	0.286
Preoperative Scr level (normal vs abnormal)	0.402	0.222–0.729	0.003

Abbreviations: AKI, acute kidney injury; OR, odds ratio; 95% CI, 95% confidence interval; MELD, model for end-stage liver disease; OLT, orthotopic liver transplantation.

## 4 Discussion

In clinical practice, synthetic colloidal solutions have been commonly used as plasma substitutes for intravascular volume supplement or volume resuscitation during OLT [23]. Given that HES may be a potential risk factor for postoperative AKI, this large-scale multicenter retrospective study evaluated the effects of intraoperative HES use on the renal function within 7 days after OLT. Although the AKIN (Acute Kidney Injury Network) classification, the RIFLE (risk, injury, failure, loss of kidney function, and end-stage renal failure) classification, and the KDIGO classification are the AKI diagnosis criteria commonly used in the clinical practice [24,25,26], we used the Scr criteria of KDIGO classification to determine the post-OLT AKI in the current study as the Scr level was a sensitive index for the early diagnosis of renal damage [27].

The overall incidence of post-OLT AKI in our study was as high as 43.7%, which is consistent with the previous literature [12,13]. However, our results showed that there was no significant difference in the incidence of post-OLT AKI among the three groups. In addition, no significant intergroup difference was noted in the 28-day mortality or the postoperative administration of RRT. Furthermore, compared with patients receiving GEL, patients receiving HES did not show any increased risk for the post-OLT AKI. Compared to patients with no HES exposure, the adjusted odds ratio for the post-OLT AKI was 0.748 (95% CI, 0.599–0.933; *P* = 0.010) in patients with low HES exposure and 1.113 (95% CI, 0.696–1.779; *P* = 0.654) in patients with high HES exposure. These results indicate that intraoperative HES use does not result in an increased risk of post-OLT AKI when compared with GEL.

Patients receiving GEL were enrolled as controls as the average molecular weight of 4% GEL is low (30 kDa), and thus, GEL can be rapidly excreted through the kidney [28]. Due to the short half-life of GEL in the blood vessel and its rapid excretion, GEL has been shown to improve the renal function in the early stage of hemorrhagic shock [29,30]. Furthermore, a meta-analysis showed that the use of a GEL solution as a plasma substitute for perioperative and critically ill patients was associated with a lower risk of acute renal failure when compared to old HES solutions with a high molecular weight [31].

The definitive mechanisms of renal toxicity caused by HES remain unknown. We speculate that the nephrotoxicity may be associated with the extravasation and deposition of HES in tissues. Osmotic nephrosis is morphologically characterized by tubular swelling due to cytoplasmic vacuole formation, and it is considered as one of the causes of renal toxicity induced by HES [32,33]. In addition, HES solutions may damage the kidney by reducing osmotic pressure [32]. With a lower degradability in the interstitial space and the reticuloendothelial system, old HES solutions with high molecular weight are inclined to accumulate, which probably contributes to the renal toxicity as well [28,33]. However, the current study provided new evidence for the kidney safety of intraoperative HES use in OLT cases.

The reasons for our results that intraoperative HES use did not increase the risk of post-OLT AKI may be multifactorial. First, OLT patients usually have a low colloid osmotic pressure due to hypoalbuminemia, and thus, the volume expansion therapy may be beneficial. In addition, the low colloid osmotic pressure may decrease the occurrence of HES extravasation. Second, the HES solution used in this study is the third generation of HES, with an average molecular weight of approximately 130 kDa and a molar substitution of approximately 0.4. The plasma clearance of HES 130/0.4 is 20-fold greater than that of old HES 450/0.7, and thus, the toxic effects of tissue deposition are lower for HES 130/0.4 [34]. Third, the volume of intraoperative HES use may be an important factor affecting the occurrence of postoperative AKT. Brunkhorst et al. found that the volume of HES was associated with the risk of AKI in a dose-effect manner [7]. A retrospective study conducted by Kashy et al. in patients with noncardiac surgery also showed that HES had dose-dependent renal toxicity [16]. Moreover, Hand et al. demonstrated that the incidence of postoperative AKI was higher in the large-volume HES group than that in the small-volume HES and albumin groups [11]. Generally, the recommended dosage of HES in clinical practice is 33 mL/kg [35], and the dosage of new-generation HES 130/0.4 is recommended as a daily maximum of 50 mL/kg [36]. In our study, the mean intraoperative dosage was only 1.7 ± 1.2 mL/kg/h in the HES group and 1.5 ± 0.9 mL/kg/h in the GEL + HES group, and the total intraoperative dosage of HES was less than 33 mL/kg. Therefore, we found that the incidence of post-OLT AKI was not significantly correlated with the HES use, which may be attributed to the relatively small dosage of HES. Fourth, the rapid recovery of liver function after OLT helps maintain hemodynamic stability and renal functions. However, septic and critically ill patients often suffer from multiple organ dysfunctions, and a relatively high dosage of HES is administrated over a prolonged period, which may increase the risk of AKI [37].

We also searched relevant studies in PubMed, EMBASE, Cochrane Library, Ovid, and a major Chinese database (CNKI). Key terms included “liver transplantation,” “hydroxyethyl starch,” “colloid,” “kidney injury,” and “renal function.” Six retrospective studies and two randomized clinical trials were retrieved, and they concluded that HES did not cause any significant postoperative renal dysfunction [10,11,23,38,39,40]. However, one of these studies showed a higher risk of AKI in patients receiving HES 130/0.4 > 30 mL/kg [39]; another study compared the effects of albumin with old HES (Hextend) on the renal outcomes after OLT and showed no significant difference in the renal function during the postoperative 30 days and 6 months, while a higher dosage of Hextend was significantly associated with worse postoperative renal function [40]. Hand et al. proposed that that intraoperative HES (130/0.4) use was associated with an increased risk of postoperative AKI in post-OLT patients when compared with albumin, which may be due to the dose-dependent renal toxicity of HES [11]. A small-scale randomized controlled trial showed that the use of HES 130/0.4 during and after OLT did not result in adverse effects on the renal function within 4 days postoperatively, while the authors also reminded prudently that their results could not be extended to patients with more severe preoperative renal injury [8]. The other randomized controlled trial performed in post-OLT patients with normal renal function found that neither GEL nor HES would cause any renal dysfunction requiring RRT, while GEL might have more remarkable renal toxicity [41].

Noteworthily, all of the aforementioned studies were small and single center. The strengths of this study are as follows: (1) a large sample size from three medical centers enhances the validity and generalizability of the results; (2) multivariable logistic regression analysis was used for controlling the potential effects of confounding variables. However, there are some limitations to the current study. First, this is a retrospective study that cannot exclude the admission bias; nevertheless, retrospective studies also have merits in the assessment of therapeutic safety as these studies have a large sample size and involve real-world patients. Second, intraoperative hemodynamic data were unavailable, which could not be included as covariates. Third, there were not enough cases for analyzing the necessity of the RRT treatment. Fourth, our grouping criteria for volumes of intraoperative HES use (none, <1,500 and >1,500 mL) are somewhat arbitrary. Finally, the incidence of post-OLT AKI during a 7-day postoperative period was evaluated, while the potential postoperative AKI-related complications were not fully investigated. In the future study, more large-scale, multicenter, randomized, and controlled clinical trials are warranted.

## 5 Conclusions

This large-scale multicenter retrospective study found that the intraoperative use of HES did not increase the overall incidence of post-OLT AKI in patients when compared with GEL, and whether to increase the risk of post-OLT AKI needs to be further explored. In addition, randomized controlled trials with large samples are warranted to further assess the kidney safety of HES in OLT patients.

## Abbreviations

HES: hydroxyethyl starch
AKI: acute kidney injury
OLT: orthotopic liver transplant
GEL: gelatin
Scr: serum creatinine
KDIGO: kidney disease improving global outcomes
RRT: renal replacement therapy
SD: standard deviation
IQR: interquartile range
OR: odds ratio
CI: confidence interval
HB: hemoglobin
MELD: model for end-stage liver disease
